# Training facilitated by interinstitutional collaboration and telemedicine: an alternative for improving results in the placenta accreta spectrum

**DOI:** 10.1016/j.xagr.2021.100028

**Published:** 2021-10-07

**Authors:** Albaro José Nieto-Calvache, José Miguel Palacios-Jaraquemada, Lina María Vergara-Galliadi, Alejandro Solo Nieto-Calvache, Maria Andrea Zambrano, Juan Manuel Burgos-Luna

**Affiliations:** 1 Clinica de Espectro de Acretismo Placentario, Fundación Valle del Lili, Cali, Colombia (Drs AJ Nieto-Calvache and Burgos-Luna); 2 Hospital Universitario, Centro de Educación Médica e Investigaciones Clínicas (CEMIC), Buenos Aires, Argentina (Dr Palacios-Jaraquemada); 3 Centro de Investigaciones Clínicas, Fundación Valle del Lili, Cali, Colombia (Dr Vergara-Galliadi); 4 Universidad Santiago de Cali, Universidad Nacional Abierta y a Distancia, Bogotá, Colombia (Dr AS Nieto-Calvache); 5 Facultad de Ciencias de la Salud, Programa de Ginecología y Obstetricia, Universidad Icesi, Cali, Colombia (Dr Zambrano)

**Keywords:** eHealth, mHealth, placenta accreta, telemedicine

## Abstract

Placenta accreta spectrum is a severe condition that requires trained, interdisciplinary group intervention. However, achieving the level of training that is required is difficult without academic programs or hospitals dedicated to teaching the necessary skills to deal with placenta accreta spectrum. We describe an interinstitutional collaboration process focused on improving placenta accreta spectrum treatment, which is facilitated by telemedicine. Lastly, we propose a replicable model for other centers.

This was a retrospective, descriptive study that included placenta accreta spectrum patients treated over a 10-year period in a low-middle income country hospital (local hospital). We evaluated the clinical results and impact of interinstitutional collaboration with a placenta accreta spectrum expert group at another low-middle income country hospital. Virtual strategies of continuous communication between the local hospital and expert group were used, such as telemedicine, teleradiology, and telepresence during surgeries.

A total of 89 placenta accreta spectrum patients were included. We observed a progressive improvement in the clinical outcomes (intraoperative bleeding, transfusion frequency, postoperative length of stay, and frequency of complications) as the fixed interdisciplinary group at the local hospital gained experience by treating more cases.

Interinstitutional collaboration (through telemedicine and remote supervision) and placenta accreta spectrum team formation were the 2 factors associated with the best outcomes in the most recent years of observation. Thus, ongoing placenta accreta spectrum team training, facilitated by interinstitutional collaboration and telemedicine, is a valid strategy for improving the clinical outcomes in placenta accreta spectrum.

## Introduction

Placenta accreta spectrum (PAS) is a disease with severe complications requiring trained interdisciplinary groups for treatment.^1^ The application of an improper surgical technique and the participation of untrained staff are factors frequently identified in the analysis of maternal deaths associated with PAS.^2^ It is recommended that groups in charge of managing this condition have extensive experience with several years of practice in treating many cases.^3^ These requirements are challenging to meet for hospitals in low- and middle-income countries (LMICs) because the custom is to treat patients in the hospital in which the diagnosis is made, and regional insurance conditions and referral protocols make it difficult to concentrate cases at certain centers.^4^ In addition, there are no specific academic programs that provide training to treat cases of PAS, and the learning curve for performing the necessary complex gynecologic-obstetrical surgery is steep.^5^

Although this problem has already been exposed, leading to calls for the development of regionalized care programs,^6^ few publications have described successful experiences in improving the training of staff at hospitals in LMICs.

We describe an interinstitutional collaborative process that facilitates PAS training by employing telemedicine and propose a model that can be replicated in other centers.

## Materials and Methods

This retrospective descriptive study included PAS patients treated between January 2011 and December 2020 at the Fundación Valle de Lili, Cali, Colombia (a local hospital [LH]). The treated population's clinical outcomes, the strategies used to improve the clinical outcomes, and the LH PAS management protocol modifications were evaluated.

Institutional review board approval was obtained (approval number 101-2020) under protocol number 929.

In mid-2015, the LH (an institution with limited experience in the management of PAS) contacted a PAS expert group (EG) in another country (CEMIC University Hospital, Buenos Aires, Argentina). After a brief face-to-face training session, an obstetrician at the LH coordinated the formation of a fixed interdisciplinary group for the management of PAS at the LH (known as the “PAS team”). This PAS team maintained continuous contact through easy-access virtual channels (phone calls, instant messaging chat, video calls through free applications) with the EG who supervised surgical performances and supported improvement initiatives at the LH.

The educational and research activities of the PAS team during the study period are also described with an emphasis on the virtual interinstitutional collaboration (LH-EG) via tele-education, teleradiology, or telemedicine methods.

Based on their experience and the observed results, the authors propose a model for institutional improvement of PAS management, which is facilitated through remote assistance.

The surgical and anesthetic protocols applied to the study population have been described previously.^7^

## Results

During the study period, 89 patients with PAS underwent surgery. Supplemental Table 1 describes the patient flow year-by-year with a more significant influx of cases since 2015 when the interinstitutional collaboration process between the LH and EG was established (Figure).

**Figure:**
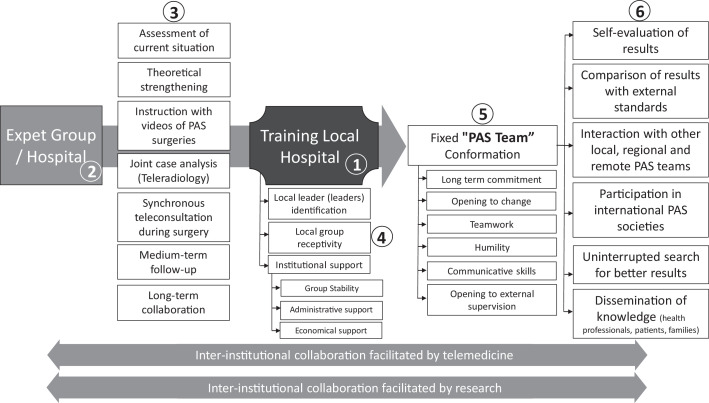
Interinstitutional collaboration model facilitated by telemedicine and research In the center of the image (1), LHs interested in improving their skills in the management of placenta accreta spectrum (PAS) can be strengthened under the mentoring or supervision of EGs in remote locations (2). Throughout the improvement process, telemedicine and research facilitate the rapprochement of groups and the sharing of information. Under remote interaction, multiple training and supervision activities are possible (3) between the training LH and EG. The LH must have the following 3 basic inputs (4): 1 or more local leader(s) interested in PAS, receptiveness of the rest of the participants in the management of PAS in the LH, and institutional support (in the form of group stability and administrative and financial support). The primary objective of the institutional improvement plan is the formation of a fixed PAS team (5) that must manifest a long-term commitment, openness to change and external supervision, communication skills, teamwork, and humility. The products of a fixed PAS team are multiple (6) and benefit not only the LH patients but also other local, regional, and remote PAS teams. *EG*, expert group; *LH*, local hospital; *PAS*, placenta accreta spectrum.

Nieto-Calvache. Training on placenta accreta spectrum facilitated by interinstitutional collaboration. Am J Obstet Gynecol Glob Rep 2021.

We observed a progressive improvement in the clinical outcomes as the LH PAS team gained experience through attending to more cases (Supplemental Table 1). In addition, there were lower frequencies of transfusions, smaller bleeding volumes, shorter postoperative hospital stays, fewer surgical complications, and a greater frequency of uterine-sparing surgery.

Supplemental Figure describes the changes that were made in the management protocol and highlights 2 factors that were identified and associated with better results:

1. Interinstitutional collaboration between an LH in search of training and an EG in another country (Figure).
2 The formation of an LH PAS team.

The PAS team had its surgical training in PAS facilitated by self-evaluation of the surgical results, the accumulation of attended cases, contact with other PAS teams in the region, and external supervision by the EG (“telepresence” during surgeries [video calls] and participation in debriefing activities).

In addition, self-evaluation and the observation of better results and contact with the EG, led to an increase in scientific productivity in the form of publications in indexed journals (Supplemental Figure).

## Discussion

### Principal findings

The PAS patients’ clinical outcomes (intraoperative bleeding, transfusion frequency, postoperative length of stay, and frequency of complications) improved year-over-year after PAS team formation. Remote assistance from an EG and the incorporation of research activities were pivotal factors in improving the clinical outcomes.

### Results

In recent years, recommendations to establish EGs in hospitals with multiple resources for the care of PAS have been reissued.^1^^,^^3^^,^^8^ However, these publications recommend conditions such as at least 5 years of experience in the care of PAS, the availability of a high number of specialties on a continuous basis (“24/7”), a constant flow of patients (2–3 cases per month, 100 cases accumulated), and complex technological resources. These requirements are difficult to meet for many LMIC hospitals that must routinely care for women with PAS and work under health insurance models that generate multiple obstacles (Supplemental Table 2).

Although the recommendations for having a high level of training to care for PAS patients and the centralization of patients in some hospitals are widely accepted, few publications have described details about the construction of regional care programs and improvement plans within a certain hospital.^6^^,^^9^^,^^10^ In addition, the solutions applied in some regions with the support of the local health system^11^ may not be valid in other locations.

PAS is more frequently being observed^12^ and is related to urinary tract lesions in 1 out of 5 patients^13^ and transfusion requirements in 3 out of 4 patients,^7^ with a risk of mortality even in industrialized countries. The main factor associated with unfavorable clinical outcomes is the lack of training of the medical teams^14^ and therefore teams that begin their training curve can benefit from the supervision and feedback of experienced teams.

Our center experienced improvements in the clinical outcomes over a 5-year period after formal interinstitutional collaboration began. We also observed better scientific production and interaction with other centers. Similar results have been reported by EGs in developed countries^15^^,^^16^ where, after generating a protocol for the treatment, including the establishment of interdisciplinary teams and incorporation of research, clinical outcomes improved.

### Clinical implications

Accepting remote assistance and identifying local leaders with institutional support seems to be a strategy that can be replicated by other institutions seeking to improve the quality of their PAS services. Experiences with interinstitutional collaboration through remote support have been reported for other rare diseases^17^ and for postpartum hemorrhage^18^^,^^19^ and PAS,^11^^,^^20^^,^^21^ all using education strategies and remote assistance as facilitators.

Although telemedicine requires formal processes and the institutions that use this strategy must build defined care routes,^22^ one of the keys to the participation of clinical professionals in virtual communities or clinical discussion groups is the ease of access to the information.^21^^,^^22^ In this sense, mHealth, taking advantage of the ubiquity of mobile phone devices and free communication platforms, offers advantages that cannot be overlooked.

### Research implications

We propose this model of interinstitutional collaboration facilitated by telemedicine and research (Supplemental Figure) as an option to improve the quality of PAS care in LMICs where the option of face-to-face training courses in other countries, visits by experts from other regions, or additional expensive resource acquisition is not an option because of economic restrictions. Therefore, additional studies evaluating the clinical impact of interinstitutional collaboration programs to improve training around PAS in other populations and with a prospective design are indispensable.

### Strengths and limitations

This study has limitations. It is a description of an experience in a single center and therefore its external validity depends on the resources available in other institutions. However, we only seek to propose an option in the absence of formal training programs in PAS and in the face of LMIC limitations.

We recognize that self-evaluation and research processes require an additional effort that many institutions cannot achieve; however, institutions that do not meet the set standards have an equally important function to promptly refer patients in need to hospitals that do.

### Conclusion

Ongoing LH PAS team training, facilitated by interinstitutional collaboration and telemedicine, is a valid strategy for improving clinical outcomes in PAS.

## Appendix Supplementary materials

Image, application 1

**Supplemental Figure**Temporal evolution of PAS healthcare (**A**) and educational (**B**) activities in a Latin American hospital under an interinstitutional collaboration model facilitated by telemedicine and research At the Fundación Valle de Lili (Cali, Colombia) patients with PAS were managed under a protocol established in 2011. However, after formal contact with an EG in another Latin American country (CEMIC, Buenos Aires, Argentina), an institutional improvement program was started that included educational activities and evaluations of surgical performance facilitated by telemedicine. The graph describes how after the interinstitutional collaboration program was established, year by year (horizontal arrow in the middle) new activities were developed to improve PAS care. Changes in the LH PAS protocol are described in the lower part of the graph (**A**). The LH management protocol began in 2011 with GA and IIAB for all patients. All patients received HT+ISP. After contact with the EG, a “PAS team” was developed in the LH, and from then on, self-evaluation activities and application of sequential changes to the surgical protocol were carried out continuously to simplify the procedure (prefer neuraxial over GA, avoiding hysterectomy in selected cases; 1-step resective reconstructive surgery, replacing IIAB with a REBOA, reserving "cell saver" for cases with massive bleeding). Since 2018, the best PAS team surgical competences have allowed the application of “intraoperative staging” to define the application of complex interventions (hysterectomy and REBOA) only in the most severe cases. In 2020 the PAS team began continuous academic interaction with other regional PAS teams and with international societies dedicated to the study of PAS (IS-PAS and PAS-2). The upper part of the graph (**B**) describes how after the beginning of the LH-EG interinstitutional collaboration around PAS (contact that is maintained to date through virtual channels), the LH academic and investigative processes were facilitated. PAS management “self-evaluation” activities were converted into a prospective research database, and the results were converted into a first scientific journal article in 2018 and several additional articles over the next 3 years. In addition, some international collaborative research has been carried out since 2020. *EG*, expert group; *GA*, general anesthesia; *HT+ISP*, total abdominal hysterectomy with “in situ” placenta; *ILAB*, internal iliac artery balloons; *IS-PAS*, International Society for Placenta Accreta Spectrum; *LH*, local hospital; *PAS*, placenta accreta spectrum; *REBOA*, resuscitative endovascular balloon occlusion of the aorta. *Nieto-Calvache. Training on placenta accreta spectrum facilitated by interinstitutional collaboration. Am J Obstet Gynecol Glob Rep 2021.* Image, application 2

Image, application 1

## Footnotes

Supplementary material associated with this article can be found, in the online version, at doi:10.1016/j.xagr.2021.100028.
