# Supplementary material for: Training facilitated by interinstitutional collaboration and telemedicine: an alternative for improving results in the placenta accreta spectrum
Source: AJOG Glob Rep. 2021 Oct 7;1(4):100028. doi: 10.1016/j.xagr.2021.100028 (PMC9563901; doi:10.1016/j.xagr.2021.100028)
Supplement: Supplementary file 1 [file mmc1.docx]

| **Supplemental Table 1. PAS patients’ clinical characteristics according to the year of observation** | | | | | | | | |
| --- | --- | --- | --- | --- | --- | --- | --- | --- |
| Variable | Patients per year | | | | | | | |
|  | 2011-2013 | 2014 | 2015 | 2016 | 2017 | 2018 | 2019 | 2020 |
| Confirmed PAS cases, n | 4 | 8 | 9 | 11 | 16 | 14 | 15 | 11 |
| IIAOB, n (%) | 3 (75%) | 8 (100%) | 8 (88.8%) | 7 (63.6%) | 3 (18.7%) | 0 | 0 | 0 |
| REBOA, n (%) | 0 | 0 | 0 | 4 (36.4%) | 13 (81.3%) | 13 (92.8%) | 9 (60.0%) | 5 (45.4%) |
| Uterine-sparing surgery, n (%) | 0 | 0 | 0 | 4 (36.4%) | 4 (25%) | 6 (42.9%) | 4 (26.7%) | 1 (9.1%) |
| Intraoperative bleeding, mL (IQR) | 1500  (ND) | 2500  (2000-3250) | 2000  (1500-2500) | 1800  (1500-2000) | 1900  (1000-2500) | 1465  (700-1894) | 1480  (619-1956) | 1394  (922-2052) |
| Transfusions frequency, n (%) | 3 (75) | 8 (100) | 5 (55) | 7 (60) | 9 (56.2) | 5 (35.7) | 6 (40) | 4 (36.4) |
| RBCU transfused, mean (IQR) | 4 (ND) | 2 (2-4) | 3 (2.5-5.5) | 4 (2-12.5) | 3.5 (2-5.2 | 2 (2-4) | 2 (1-2) | 1.5 (1-2) |
| Postoperative hospitalization (days) | 16 (6-27) | 4 (3-8) | 4 (2-5) | 4 (3-7) | 2 (2-3) | 2 (2-3) | 3 (2-4) | 3 (2-4) |
| Complications, n (%) * | 3 (75) | 4 (50%) | 4 (44.4) | 4 (36.4) | 6 (37.5) | 4 (28.5) | 4 (26.6) | 4(36.4) |
| PAS: placenta accreta spectrum. IIAOB: internal iliac artery occlusion balloon. REBOA: resuscitative endovascular balloon occlusion of the aorta. ND: no data (not enough data to calculate). IQR: interquartile range. RBCU: red blood cells units.  * Any surgery-related complication (endovascular device-related complications, bladder rupture, ureteral injury, requirement of a second surgery). | | | | | | | | |

| **Supplemental** **Table 2. Obstacles faced by LMIC hospitals to become centres of excellence in PAS** |
| --- |
| 1. They receive a low number of PAS cases each year since not all patients with PAS diagnoses can be referred to these centres due to administrative limitations, or there are multiple similar centres in the region that “compete” for PAS care. 2. They do not have a fixed group of specialists because the care of their users is in charge of the "doctors on duty" every day, without the possibility of recognizing medical fees for additional "on call" personnel. This delays the time required to overcome the "training curve". 3. Although they have a high flow of PAS patients and extensive surgical training, the work overload makes it difficult to self-evaluate and implement PAS-related educational and research activities. 4. Although they receive a high number of PAS patients and have trained surgeons, they lack the technological and human resources recommended by international consensus on PAS (cell saver, interventional radiology, multiple subspecialties of immediate availability "24 x 7", etcetera). 5. They are forced to provide care to PAS patients, even if they do not have the recommended resources, since they are the only institution in the region or they do not have hospitals to refer to (due to insurance problems or geographical limitations). |
| LMIC: low- and middle-income countries. PAS: placenta accreta spectrum. |
