# Supplementary material for: Training facilitated by interinstitutional collaboration and telemedicine: an alternative for improving results in the placenta accreta spectrum
Source: AJOG Glob Rep. 2021 Oct 7;1(4):100028. doi: 10.1016/j.xagr.2021.100028 (PMC9563901; doi:10.1016/j.xagr.2021.100028)
Supplement: Supplementary file 2 — Supplemental FigureTemporal evolution of PAS healthcare (A) and educational (B) activities in a Latin American hospital under an interinstitutional collaboration model facilitated by telemedicine and research At the Fundación Valle de Lili (Cali, Colombia) patients with PAS were managed under a protocol established in 2011. However, after formal contact with an EG in another Latin American country (CEMIC, Buenos Aires, Argentina), an institutional improvement program was started that included educational activities and evaluations of surgical performance facilitated by telemedicine. The graph describes how after the interinstitutional collaboration program was established, year by year (horizontal arrow in the middle) new activities were developed to improve PAS care. Changes in the LH PAS protocol are described in the lower part of the graph (A). The LH management protocol began in 2011 with GA and IIAB for all patients. All patients received HT+ISP. After contact with the EG, a “PAS team” was developed in the LH, and from then on, self-evaluation activities and application of sequential changes to the surgical protocol were carried out continuously to simplify the procedure (prefer neuraxial over GA, avoiding hysterectomy in selected cases; 1-step resective reconstructive surgery, replacing IIAB with a REBOA, reserving "cell saver" for cases with massive bleeding). Since 2018, the best PAS team surgical competences have allowed the application of “intraoperative staging” to define the application of complex interventions (hysterectomy and REBOA) only in the most severe cases. In 2020 the PAS team began continuous academic interaction with other regional PAS teams and with international societies dedicated to the study of PAS (IS-PAS and PAS-2). The upper part of the graph (B) describes how after the beginning of the LH-EG interinstitutional collaboration around PAS (contact that is maintained to date through virtual channels), the LH academic and investigative pro [file mmc2.pptx]

## Slide 1
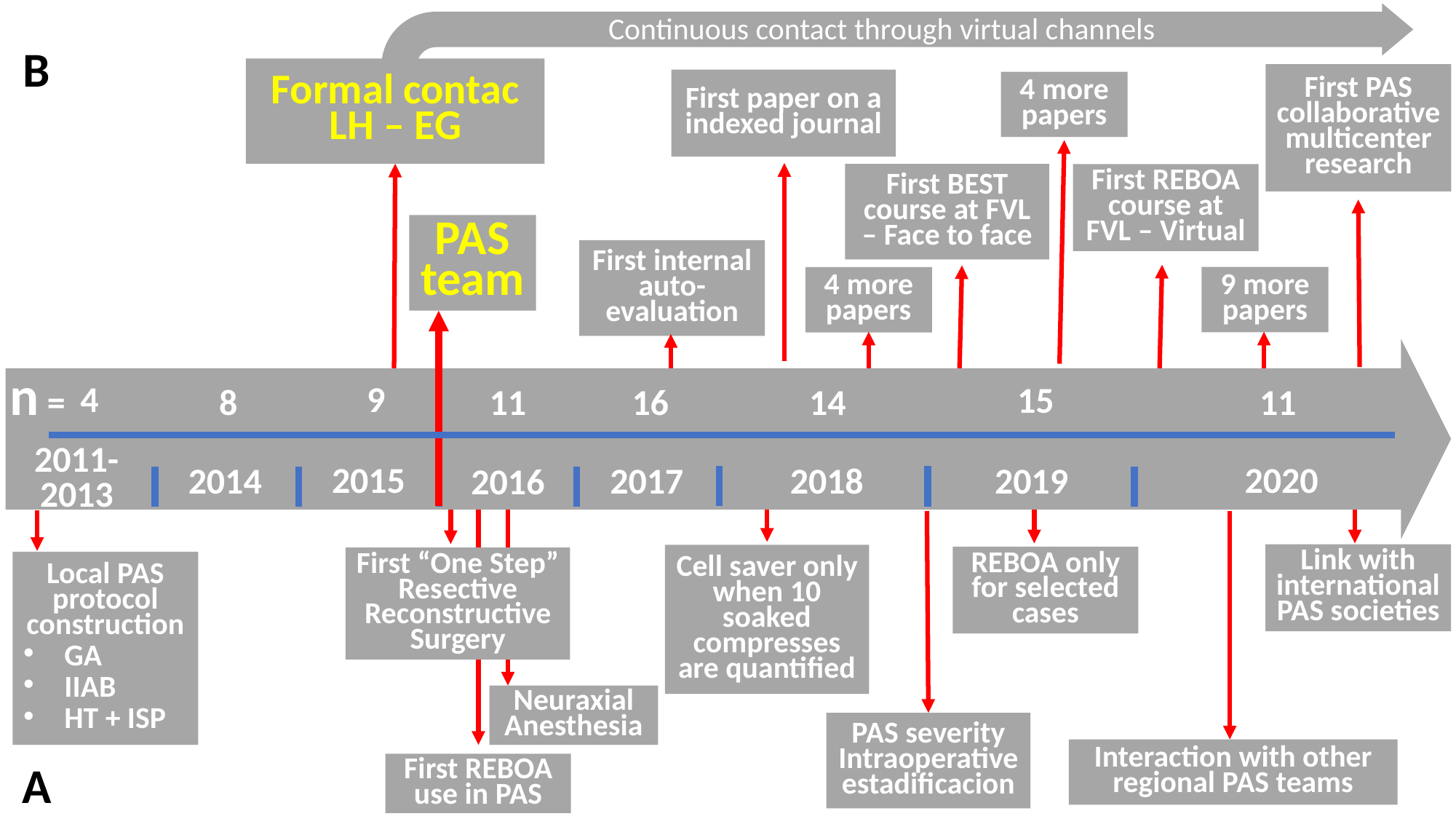

Continuous contact through virtual channels
B
Formal contac LH – EG
First PAS collaborative multicenter research
First paper on a indexed journal
4 more papers
First BEST course at FVL – Face to face
First REBOA course at FVL – Virtual
PAS team
First internal auto- evaluation
9 more papers
4 more papers
n =
4
9
15
8
11
16
14
11
2011-
2013
2015
2020
2014
2017
2018
2019
2016
Link with international PAS societies
Cell saver only when 10 soaked compresses are quantified
REBOA only for selected cases
First “One Step” Resective Reconstructive Surgery
Local PAS protocol construction
GA
IIAB
HT + ISP
Neuraxial Anesthesia
PAS severity Intraoperative estadificacion
Interaction with other regional PAS teams
First REBOA use in PAS
A
