# Supplementary material for: Training facilitated by interinstitutional collaboration and telemedicine: an alternative for improving results in the placenta accreta spectrum
Source: AJOG Glob Rep. 2021 Oct 7;1(4):100028. doi: 10.1016/j.xagr.2021.100028 (PMC9563901; doi:10.1016/j.xagr.2021.100028)

Supplementary material 2. Photos of each case with One Step Conservative Surgery. **Case 1. Bolivia. Hospital 1**

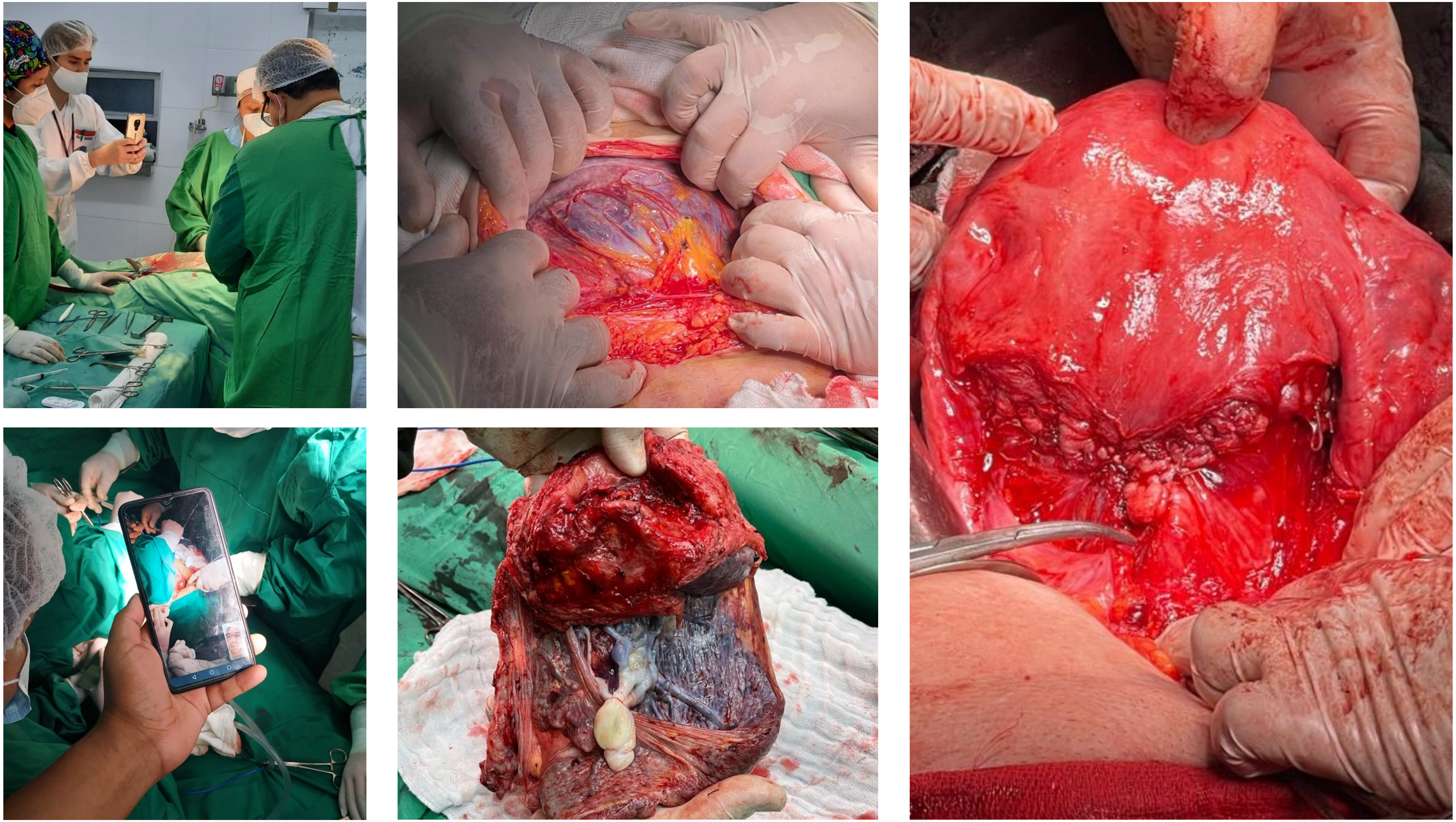

Supplementary material 2. Photos of each case with One Step Conservative Surgery. **Case 2. Bolivia. Hospital 2**

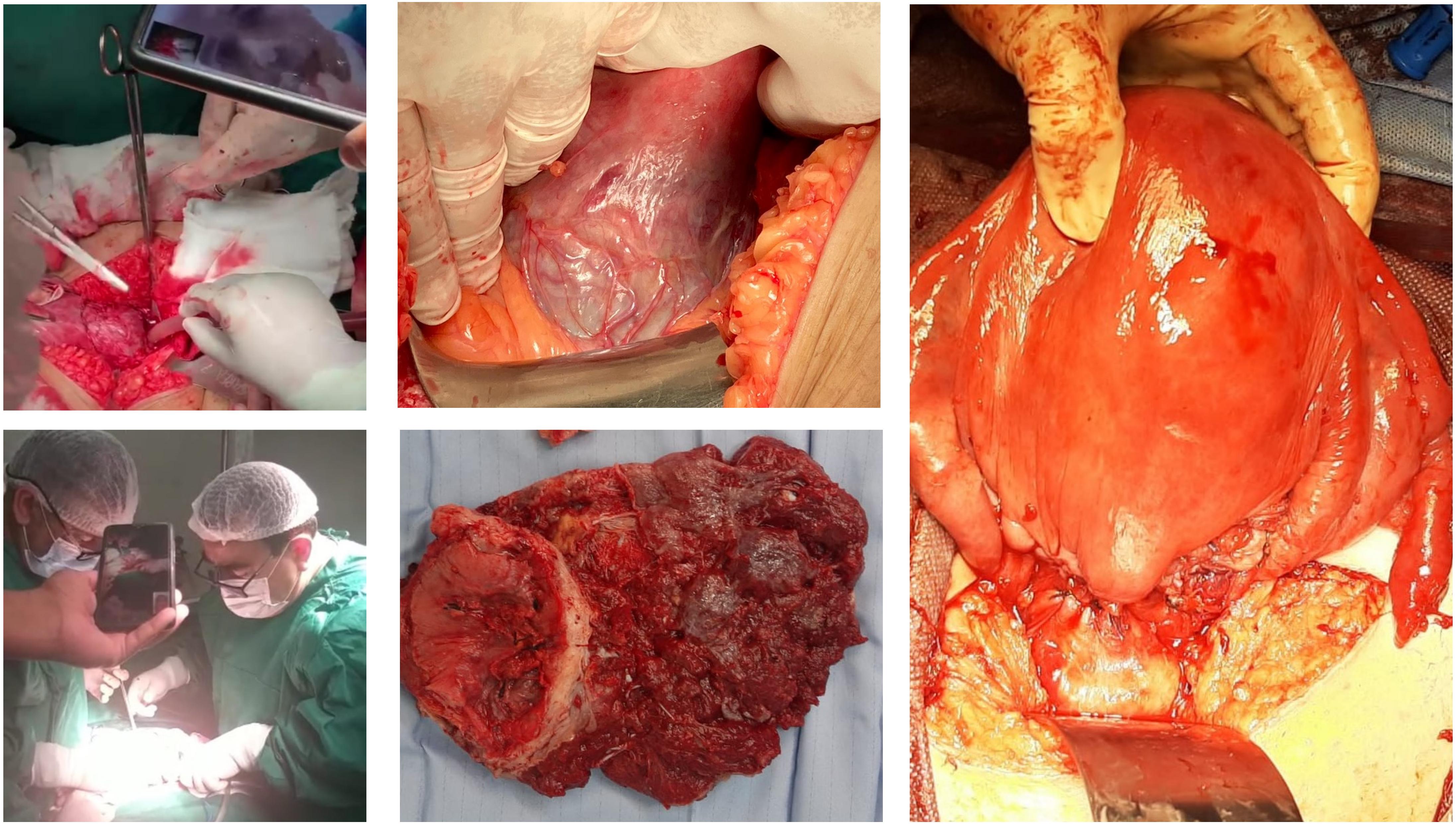

Supplementary material 2. Photos of each case with One Step Conservative Surgery. **Case 3. Guatemala**

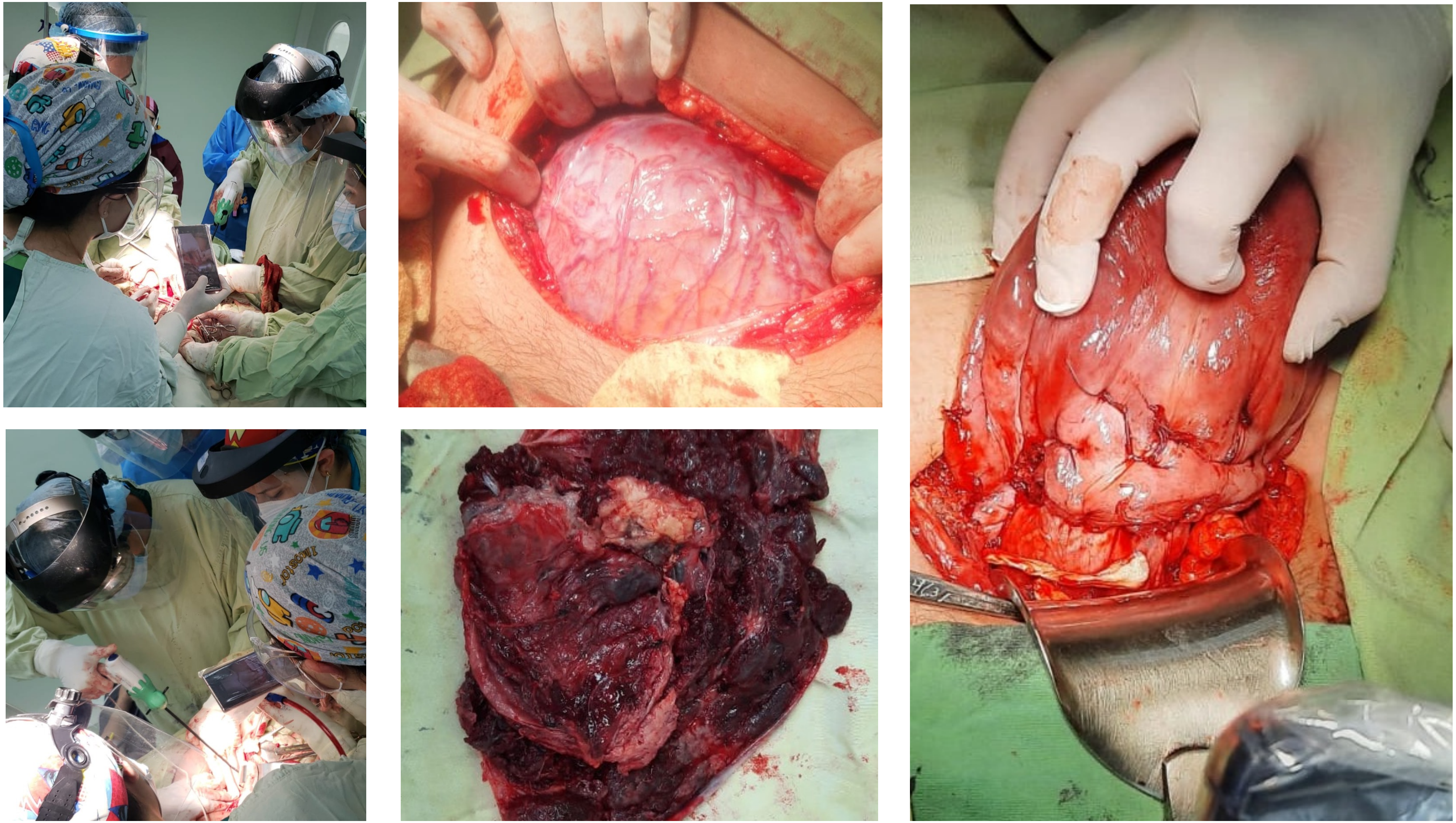

Supplementary material 2. Photos of each case with One Step Conservative Surgery. **Case 4. Colombia. Hospital 1**

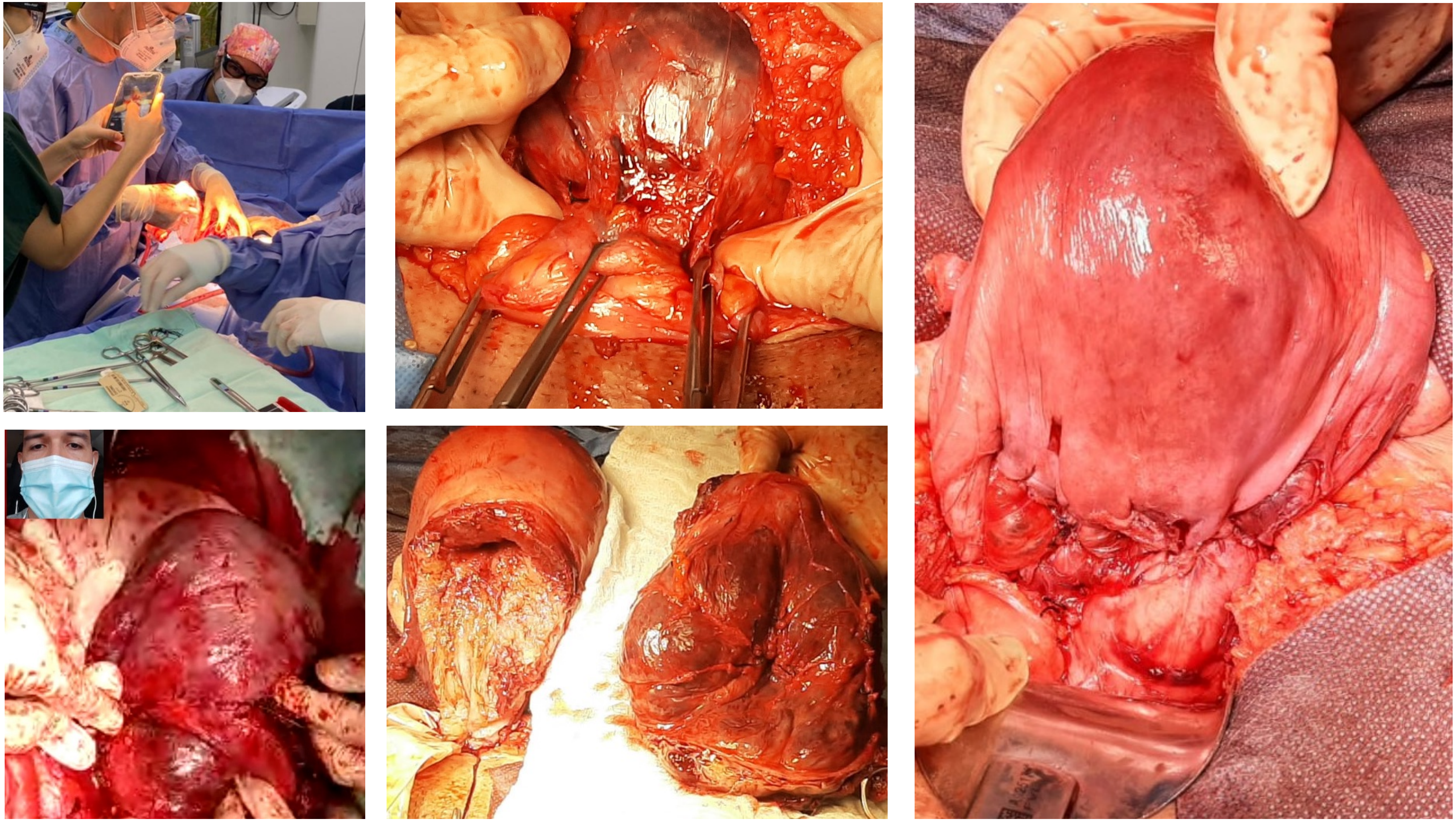

Supplementary material 2. Photos of each case with One Step Conservative Surgery. **Case 5. Colombia. Hospital 2**

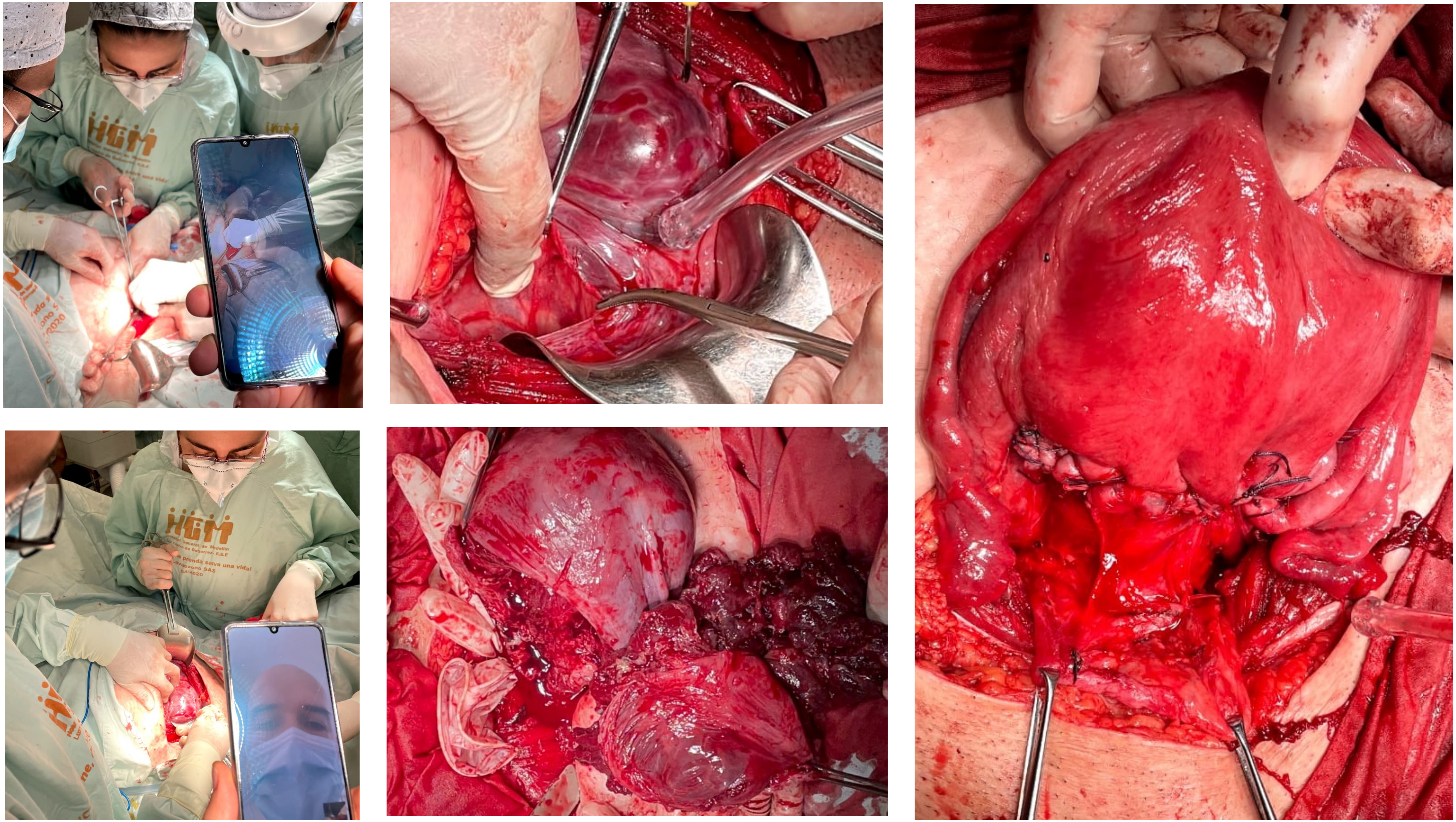

Supplementary material 2. Photos of each case with One Step Conservative Surgery. **Case 6. Indonesia**

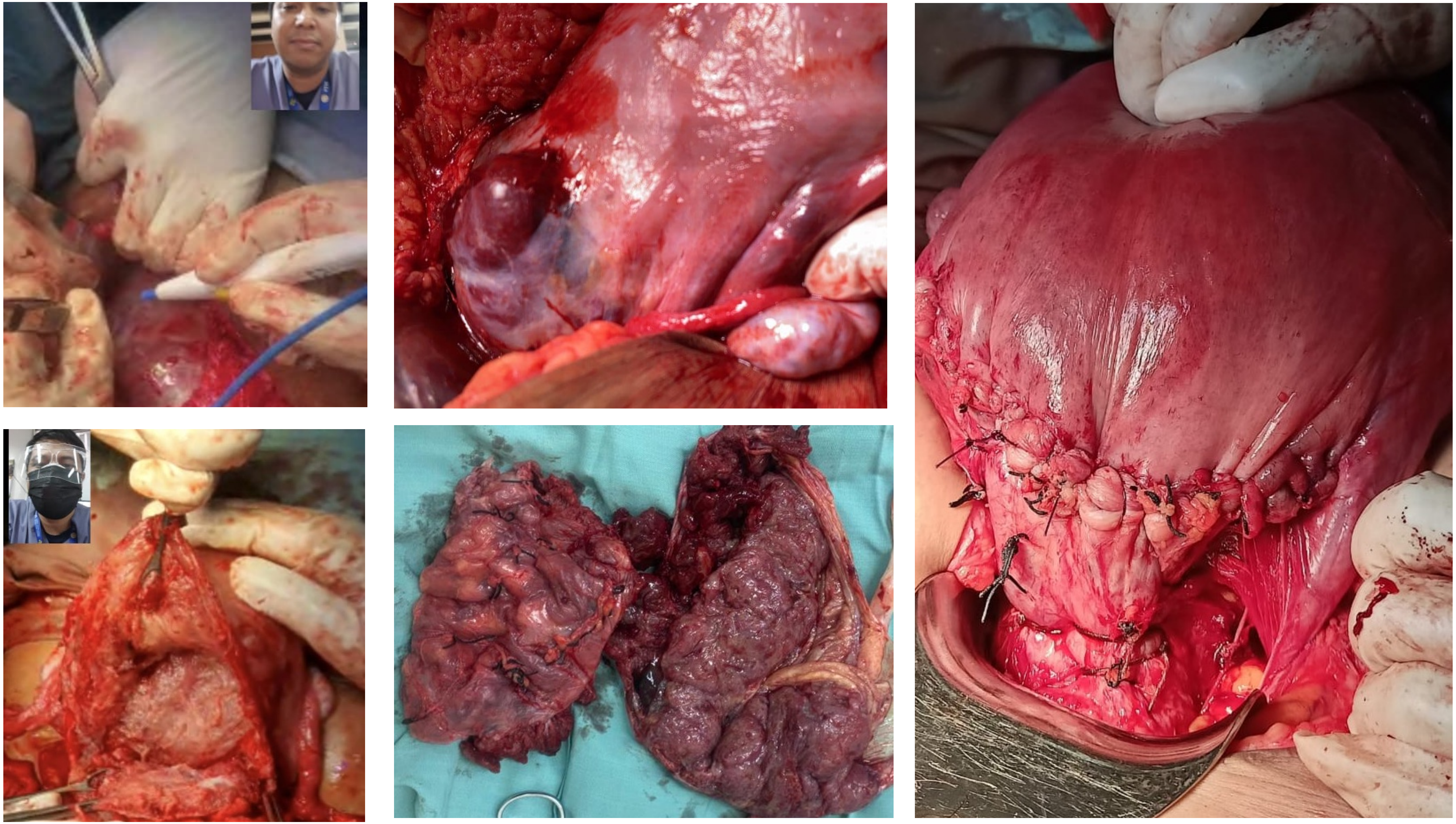

Supplement: Supplementary file 3 [file mmc3.pdf]
